# Nanocomposite–parylene C thin films with high dielectric constant and low losses for future organic electronic devices

**DOI:** 10.3762/bjnano.10.42

**Published:** 2019-02-12

**Authors:** Marwa Mokni, Gianluigi Maggioni, Abdelkader Kahouli, Sara M Carturan, Walter Raniero, Alain Sylvestre

**Affiliations:** 1 Univ. Grenoble Alpes, CNRS, Grenoble INP, G2Elab, 38000 Grenoble, France; 2 Dipartimento di Fisica e Astronomia “G. Galilei”, Università di Padova, Via Marzolo 8, 35121 Padova (PD), Italy; 3 Istituto Nazionale di Fisica Nucleare, Laboratori Nazionali di Legnaro, Viale dell’Università 2, 35020 Legnaro (PD), Italy

**Keywords:** dielectric, nanocomposite polymer, organic field-effect transistor, parylene C, silver-containing nanoparticle

## Abstract

Nanocomposite–parylene C (NCPC) thin films were deposited with a new technique based on the combination of chemical vapor deposition (CVD) for parylene C deposition and RF-magnetron sputtering for silver deposition. This method yields good dispersion of Ag-containing nanoparticles inside the parylene C polymer matrix. Film composition and structure were studied by using several techniques. It was found that the plasma generated by the RF-magnetron reactor modifies the film density as well as the degree of crystallinity and the size of parylene C crystallites. Moreover, silver is incorporated in the parylene matrix as an oxide phase. The average size of the Ag oxide nanoparticles is lower than 20 nm and influences the roughness of the NCPC films. Samples with various contents and sizes of silver-oxide nanoparticles were investigated by broadband dielectric spectroscopy (BDS) in view of their final application. It was found that both the content and the size of the nanoparticles influence the value of the dielectric constant and the frequency-dependence of the permittivity. In particular, β-relaxation is affected by the addition of nanoparticles as well as the dissipation factor, which is even improved. A dielectric constant of 5 ± 1 with a dissipation factor of less than 0.045 in the range from 0.1 Hz to 1 MHz is obtained for a 2.7 µm thick NCPC with 3.8% Ag content. This study provides guidance for future NCPC materials for insulating gates in organic field-effect transistors (OFETs) and advanced electronic applications.

## Introduction

Increasing the dielectric constant of gate dielectrics for oxide thin-film transistors (TFTs) improves the performance of such devices. Challenges are in the processing of these high-*k* dielectrics and various approaches were tested over time. Among them, low-cost and innovative methods were recently proposed for low operating voltages of TFTs [1–2]. By using water-inducement, scandium oxide was succesfully integrated as gate dielectric in both InZnO and CuO TFTs [1]. Using a sol–gel approach, high-*k* ink hybrid AlOOH nanocomposites demonstrated low leakage currents suitable for low operating voltages of TFTs [2]. Unfortunately these approaches can not be used when parylene C (PPXC) is chosen as gate dielectric as the only proven process for producing high-quality PPXC layers is chemical vapor deposition (CVD). Parylene C has emerged as a particularly interesting material for organic electronic devices as a gate dielectric, coating insulator film, or flexible substrate [3–5] due to its numerous advantageous properties. PPXC films are biocompatible and environmentally friendly [6–9]. Its deposition process makes it accessible as a coating for many semiconductor polymers [10] for organic field-effect transistors (OFETs) [11], organic light-emitting diodes (OLEDs) [12–13], and flexible organic electronic devices (FEDs) [14–15]. It presents an easy deposition process at low temperatures with a conformal and uniform layer [16]. Parylene C is a well-controlled material when used as gate dielectric, which is a crucial requirement for the performance of the OFETs and for the device reliability. Charge-carrier mobility is improved in the presence of this polymer [17]. PPXC is also an appropriate hydroxyl-free gate dielectric and prevents trapping of electrons at the semiconductor–dielectric interface in contrast to polymers containing hydroxyl groups such as poly(vinyl phenol) and polyimides (due to residual COOH groups) [18–20]. The stability of the devices, which is impacted by this charge trapping at the interfaces, is improved when parylene C is integrated in the device [21–22]. Parylene C is highly corrosion resistant on metallic surfaces and possesses outstanding electrical insulation with high tensile strength, moderate dielectric losses [16,23] and low permeability to gases [24–25]. Hydrophobicity [26] and physical stability [27] of parylene C make it a good candidate as a coating dielectric material to protect the sensitive organic layer from oxygen and water vapor [28], which are among the greatest degradation mechanisms contributing to the electrical instability of OFETs [29–32] and oxide TFTs [33].

It is inferred that parylene C presents a broad applicability and a versatile role in the technology of OFETs and organic compounds [3]. However, parylene C, as the vast majority of polymers, exhibits a low dielectric constant (3.15 at 1 kHz [34]) thus limiting its performance in specific applications in OFETs and electronic devices.

Using nanocomposite polymers as gate dielectrics presents several advantages for the improvement of the electronic device properties such as higher dielectric constant [35] and dielectric strength [36], reduced threshold voltage [37], increased charge mobility and reduced leakage current [38]. Compared to pure parylene C and other pure materials such as SiO_2_, polyimide, polyethylene, alumina (Al_2_O_3_), benzocyclobutenes (BCB) and SiO_2_/poly(methyl methacrylate) (PMMA), nanocomposite parylene C (NCPC) exhibits some interesting properties [39–47]. As an example, parylene C/Silica nanocomposites show greatly improved mechanical properties and thermal stability in comparison to pure PPXC films [48]. In a recent study, these properties, and especially thermal and UV stability, were further improved by combining nanosilica/titania particles with parylene C [49]. As shown in other works [50–51], parylene C/Al_2_O_3_ bilayers applied to medical devices exhibit a longer-term reliability in comparison to pure PPXC.

The goal of this study is to improve the electrical properties of parylene C used in advanced electronic devices [52–54] as a gate dielectric or an insulation coating. The challenge is to increase the dielectric constant of NCPC without degrading its dielectric losses. In this context, this work presents a new strategy to synthesize nanocomposite parylene C materials by a combination of two processes, CVD and RF-magnetron sputtering. The NCPC properties are analyzed in detail by different experimental techniques. Particularly, in order to evaluate the effect of the Ag-containing nanofiller charges regarding a possible integration as gate insulating material for OFETs, broadband dielectric spectroscopy (BDS) is carried out on NCPCs with different content of silver-containing nanoparticles. As a final result, a gain in the gate insulation capacitance is expected for OFETs with the addition of conductive particles inside the native parylene C insulating gate.

## Results and Discussion

### Silver-PPXC co-deposition: film composition and structure

Table 1 gives the experimental parameters of deposited pure parylene C and NCPCs. Apart from sample O, which was produced by keeping the sputtering source off, all the samples were deposited with the plasma switched on but with different numbers of rotations with the shutter open (i.e., changing the amount of Ag atoms incorporated inside the film). Therefore, the number of rotations increases from 0 (sample K) to 6 (sample F). Samples from A to F are multilayers (three layers), consisting of a pure parylene C layer (PPXC, 1st layer) followed by an Ag-containing parylene C layer (PPXC+Ag, 2nd layer) and then by another pure parylene C layer (PPXC, 3rd layer). The thickness of each single layer (either with or without Ag) was measured by RBS in monomeric units·cm^−2^. As highlighted in the Experimental section (see below), the parylene C amount deposited on the substrate (in monomeric units·cm^−2^) is directly obtained from the Cl RBS atomic dose, since each monomeric unit contains one Cl atom. In the case of three-layered samples (from A to F), the SIM simulation package in the RUMP software [55] was used to simulate the experimental spectra and to determine the thickness of each layer (in monomeric units·cm^−2^).

**Table 1:** Experimental parameters of pure parylene C and NCPCs deposited by combined CVD and RF sputtering at room temperature. The layer sequence starts from the sample surface. The single layer thickness is measured in 10^17^ monomeric units·cm^−2^. The total Ag content has been calculated by dividing the Ag dose (in atoms·cm^−2^) by the total parylene amount (in monomeric units·cm^−2^). The total thickness (in µm, last column) was measured by a mechanical profilometer.

sample	RF power to Ag target (W)	layer sequence from surface: 1. 1st layer 2. 2nd layer 3. 3rd layer	thickness of each single layer (10^17^ monomeric units·cm^−2^)	total Ag content (%)	total thickness (µm)

O	NO	PPXC	4.4	—	0.75 ± 0.05
K	60	PPXC	6.4	—	1.32 ± 0.06
A	120	1. PPXC 2. PPXC+Ag (1 rotation) 3. PPXC	1. 3.4 2. 0.75 3. 4.3	1.0	1.72 ± 0.03
B	120	1. PPXC 2. PPXC+Ag (1 rotation) 3. PPXC	1. 4.4 2. 0.75 3. 3.6	1.8	1.76 ± 0.04
C	120	1. PPXC 2. PPXC+Ag (2 rotations) 3. PPXC	1. 4.6 2. 2.2 3. 5.0	3.8	2.68 ± 0.14
D	120	1. PPXC 2. PPXC+Ag (3 rotations) 3. PPXC	1. 5.2 2. 2.8 3. 2.7	2.6	2.07 ± 0.07
E	120	1. PPXC 2. PPXC+Ag (4 rotations) 3. PPXC	1. 4.0 2. 4.8 3. 3.3	2.5	2.49 ± 0.05
F	120	1. PPXC 2. PPXC+Ag (6 rotations) 3. PPXC	1. 5.5 2. 6.0 3. 0.20	4.45	2.30 ± 0.04

**Figure 1:**
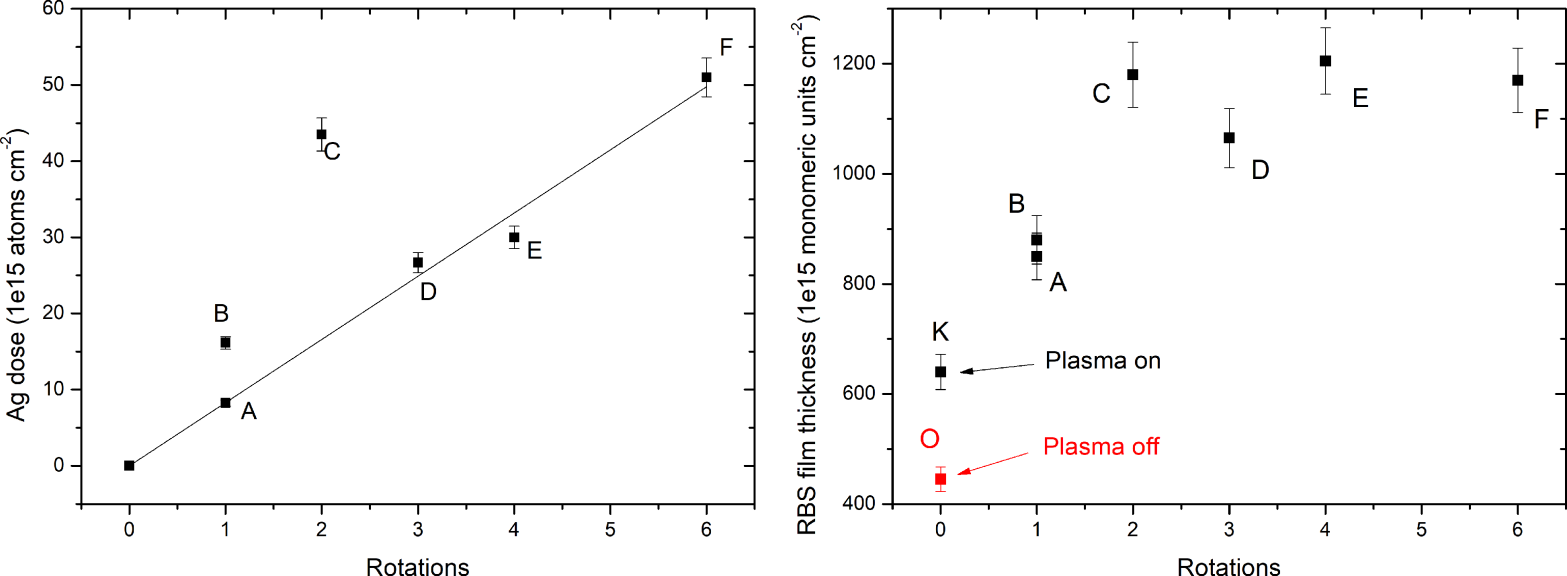
Ag dose (left) and film thickness (right) versus rotations.

Figure 1 (left) shows the corresponding Ag dose incorporated in these samples as a function of the number of rotations. If the samples B and C are neglected, one finds that the dose increases almost linearly as could be expected. Ag dose incorporated during a single rotation is 8.3 × 10^15^ atoms·cm^−2^. The reason of the anomalous behavior of samples B and C, the dose of which is higher than expected, is not completely clear, but it is thought to lie mainly in the very peculiar nature of the parylene deposition process, which is controlled by setting the pressure inside the chamber. This control method gives rise to hysteresis loops in the chamber pressure and then in the crucible temperature, which can last for several minutes. The loops can then produce an oscillation in the parylene sublimation rate during the film deposition. If the Ag deposition coincides with a low parylene deposition rate, the parylene deposition on the Ag target surface will be lower and then the Ag sputtering yield will increase together with the Ag content incorporated in the film. The Ag incorporation will be further enhanced, if one considers that the lower the parylene deposition rate, the lower the pressure in the chamber, the higher the number of Ag atoms reaching the substrates. The anomalous behavior of samples B and C highlights the complexity of this new deposition method, which arises from coupling two different processes (CVD and sputtering) for the first time. This process complexity is also responsible for the very different thickness of 1st and 3rd layers in some of the NCPCs (see, e.g., samples F and D). In order to solve this problem, a process control based on setting a direct parameter (such as, e.g., parylene deposition rate) instead of the total pressure in the chamber would be more effective.

Figure 1 (right) shows the film thickness measured in monomeric units·cm^−2^ (by RBS) of the samples O, K and from A to F as a function of the number of rotations. The first important feature to be noted is the effect of plasma on the deposition rate of parylene. When the plasma is switched on (sample K), we observe an increase of the amount of parylene deposited on the substrate compared to when the plasma is switched off (sample O). This increase is thought to be due to an increase of the turbulent motions inside the chamber, which perturb the flow of monomer molecules, resulting in an increase of the parylene residence time, as already observed in the case of co-deposition of UV absorber and parylene [56]. When the shutter is open, the deposition rate further increases (samples A, B and C) showing that the plasma effect on the monomer flow is more pronounced. The deposition rate reaches a plateau for the remaining samples (see samples C to F).

**Figure 2:**
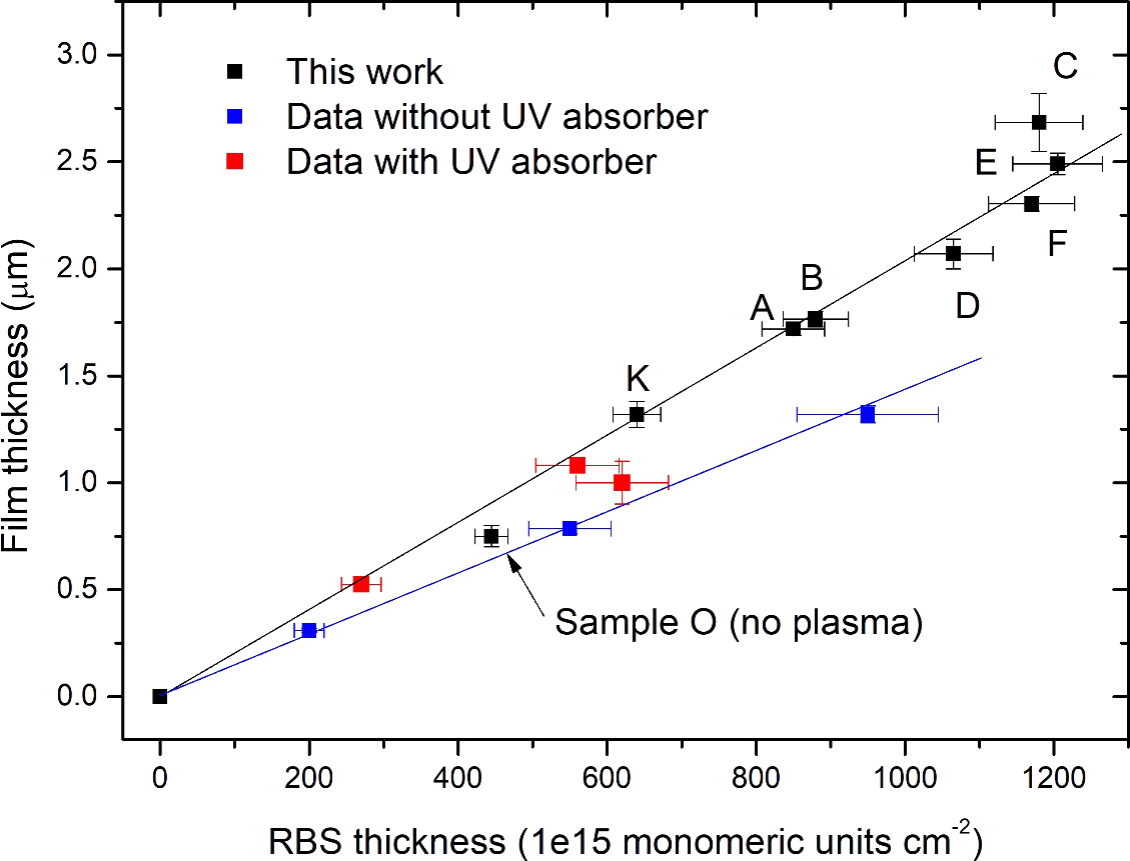
Film thickness versus RBS thickness (see text for details). Blue and red squares data are drawn from [56]. The two lines are guides for the eye.

The plasma-induced increase of the deposition rate affects the film density, as shown by the data in Figure 2, where the film thickness measured in micrometers (by the profilometer) is plotted as a function of the thickness measured in monomeric units·cm^−2^ (by RBS). In Figure 2 the data for our set of samples (black squares) are compared with those of parylene samples deposited without plasma (blue squares, from [56]) and those without plasma but with co-deposition of UV-absorber (red squares, from [56]). Figure 2 highlights the good linear correlation between film thickness measured by profilometer and RBS for both sets of samples with and without plasma (black and blue squares, respectively) up to a value of about 1.0 × 10^18^ monomeric units·cm^−2^, as highlighted by the two straight lines. Moreover, the different slopes for these two data sets imply that, for the same RBS thickness, the samples deposited with plasma have a higher physical thickness. This means that the plasma gives rise to a decrease of the film density, as already found in the case of samples obtained by co-deposition of parylene and UV-absorber (red squares), the thickness of which is closer to that of plasma-deposited samples.

**Figure 3:**
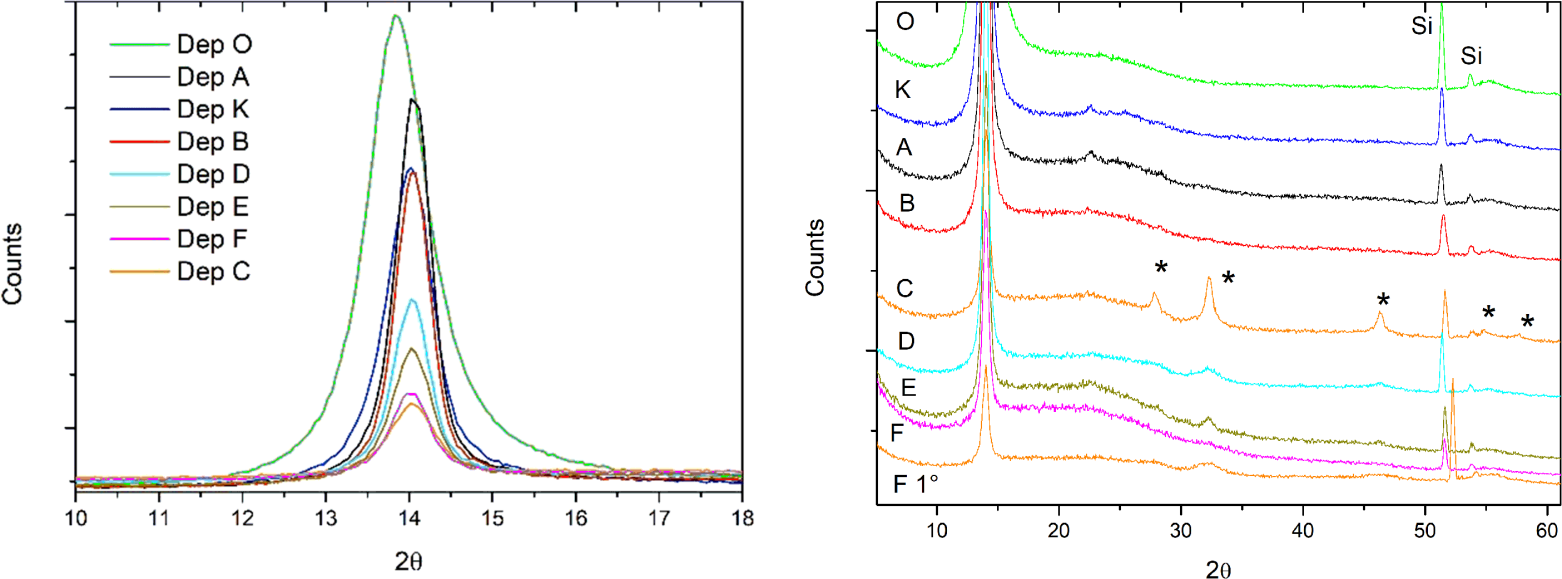
GIXRD spectra of pure and NCPC samples: left) the 2θ region of the main parylene peak; right) the entire 2θ range, which shows the effect of Ag incorporation on the structure of the films. The spectrum of sample F at 1° incidence angle is also shown. Asterisks indicate the peaks of Ag oxides. Si peaks coming from the substrate are also highlighted.

The decrease of film density matches the change of structural order in the film matrix, as shown by GIXRD results (Figure 3). Spectrum of film deposited when plasma is off (sample O) shows only the characteristic reflection at 2θ = 13.85° ((020) plane of a monoclinic unit cell with dimensions: *a* = 5.96 Å, *b* = 12.69 Å, *c* = 6.66 Å, β = 135.28° [57]), thus highlighting the strong preferred orientation of the parylene nanocrystalline domains in this sample. When the plasma is switched on, we observe that the (020) peak becomes less intense in spite of the higher parylene amount and shifts to 2θ = 14.00–14.05°. The average parylene nanocrystallite size, as determined through the Scherrer equation [58] applied to this peak, slightly increases from around 9 nm for sample O to 12–16 nm for the plasma-deposited samples (see Table 2 for the FWHM values of the (020) peak used for the calculation of the average size). Moreover, another peak at 2θ = 22.33°, assigned to the (110) parylene reflection, appears, even if very weakly in samples C to F. All these features hint at a different structural arrangement of the parylene matrix in the plasma-deposited samples, consisting in a lower preferred orientation and a higher amount of randomly oriented nanocrystalline domains. It is inferred that this evolution is mainly an effect of the plasma, while Ag incorporation plays only a minor role.

When silver deposition is enabled (open shutter), peaks of Ag-containing nanocrystallites appear and are clearly visible in the spectrum of sample C at 2θ = 27.8°, 32.3°, 46.3°, 54.9° and 57.6°. All these peaks can be referred to silver-oxide phases, i.e., Ag_2_O [59], Ag_3_O_4_ [60], AgO [61], Ag_2_O_2_ [62] and Ag_2_O_3_ [63]. It is noteworthy that there is no peak that can be ascribed to metal Ag phases. Taking into account that Ag is sputtered from the target surface as metal atoms, it is thought that the silver oxidation occurs mostly during the film growth owing to the relatively high residual pressure (2–3 Pa) in the deposition chamber, which promotes the adsorption and incorporation of oxygen-containing species (such as oxygen and water vapor molecules) in the growing film. On the other hand, post-deposition silver oxidation in the external environment can not be completely ruled out, because the lower density of plasma-deposited films can decrease the well-known gas barrier properties of parylene C and then promote the diffusion of oxygen-containing species inside the films. In order to decrease the oxidation, the residual pressure in the chamber should be drastically reduced (e.g., using a high-vacuum pump). Concerning the silver-oxide peaks, the reason why they are much more visible in the spectrum of sample C than in the spectra of the other samples can be easily understood if one considers the properties of these samples. In sample C, the Ag dose is very high (see Figure 1, left) and concentrated in a thin parylene layer (2.2 × 10^17^ monomeric units·cm^−2^, see Table 1). Hence, the formation of nanocrystallites with higher average size is promoted (the size is around 13–14 nm, as determined through the Scherrer equation applied to the most intense peak at 32.3°; see Table 2 for the FWHM values used in the size calculation). On the other hand, weaker peaks are visible in samples D and E because Ag dose is lower than that of sample C and is distributed in a thicker layer (2.8 × 10^17^ and 4.8 × 10^17^ monomeric units·cm^−2^, respectively) so that smaller nanocrystallites grow (average sizes of 6–7 nm and 10–11 nm, respectively).

For samples A and B, which do not show any peaks, we had to increase the acquisition time due to the low total Ag dose in these samples and then an average size of 6–7 nm was found for both samples. In the case of sample F, which has the highest Ag dose, the Ag-containing layer is buried below a thick parylene layer (see Table 1) so that we had to increase the X-ray incidence angle to 1.0° in order to probe all the film thickness and highlight the Ag oxides crystalline peaks (see Figure 3 right). The broadness of these peaks indicates that the average nanocrystallite size is small (≤5 nm), as could be expected taking into account that silver is distributed in an even thicker layer (6.0 × 10^17^ monomeric units·cm^−2^) as compared to the other samples. As a general remark, the small average size of Ag-containing nanoparticles (less than 20 nm for all the samples) confirms their fine dispersion in the parylene matrix, already highlighted in a previous work [64].

**Figure 4:**
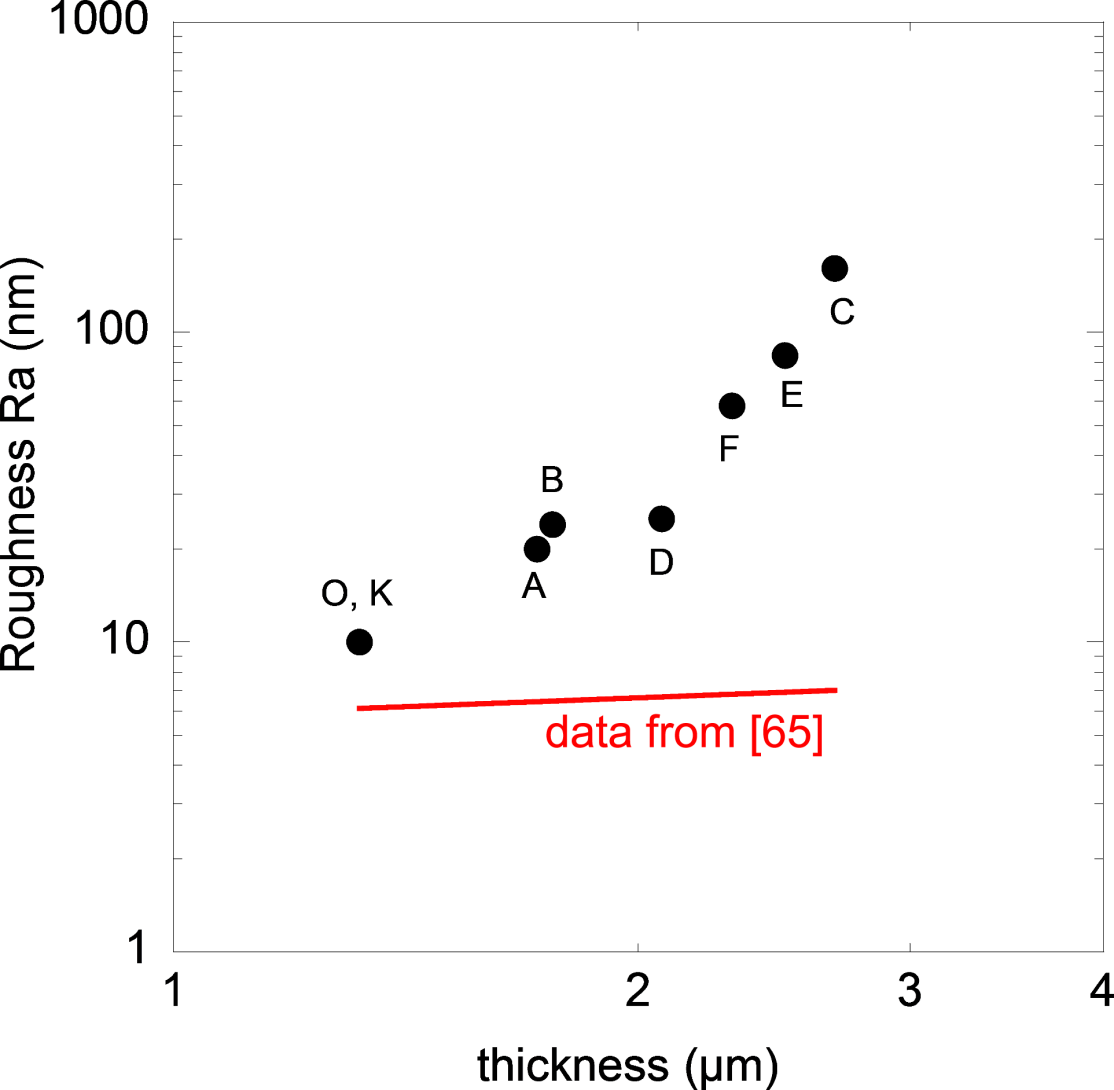
Roughness (Ra) as a function of the thickness. The red curve is the trend for pure parylene C films obtained in [65].

The roughness of different NCPC samples was measured by AFM and displayed in Table 2. In the case of pure parylene films (samples K and O), the surface is relatively smooth and the roughness is around 10 nm. It has to be noted that the plasma-induced increase of the deposition rate does not change the film roughness. When Ag is incorporated in the parylene matrix, the films become increasingly rough with increasing Ag amount, the roughness increasing from 20–25 nm for samples A, B and D to more than 50 nm for samples C, E and F. Even if it has been previously shown that an increase in parylene C thickness produces an increase in roughness [65], the increase for the pure parylene C samples in [65] is much less pronounced as compared to our samples, as can be appreciated in Figure 4, which shows the trend of the two sets of data. Therefore, the thickness does not appear to be the main criterion explaining the roughness in our NCPC samples. It is inferred that maybe there is also an effect of nanoparticle size because the highest roughness is found in samples E and C, which have also the greatest average nanoparticle size (10–11 nm and 13–14 nm, respectively).

**Table 2:** XRD data and AFM roughness of NCPC samples: effect of Ag incorporation on the peak width (FWHM) and roughness (Ra, Rq) of the films.

sample	O	K	A	B	C	D	E	F

FWHM of parylene peak	0.89	0.67	0.52	0.51	0.64	0.53	0.57	0.57
FWHM of AgO*_x_* peak	—	—	1.35	1.20	0.61	1.30	0.77	1.9
Ra (nm)	10	10	20	24	161	25	84	58
Rq (nm)	14	13	30	36	201	35	106	75

The total Ag content appears to play a minor role as compared to the nanoparticle size, as shown by the samples F and E. The former has the highest Ag content and the lowest nanoparticle size and ultimately exhibits a lower roughness than the samples E and C. The latter, with an Ag content equivalent to that of sample D, has a greater roughness than sample D maybe due to a larger nanoparticle size. To summarize, film thickness, AgO*_x_* nanoparticle size and, to a lesser extent, Ag content concur to affect the roughness of the NCPC samples.

### FTIR analysis

**Figure 5:**
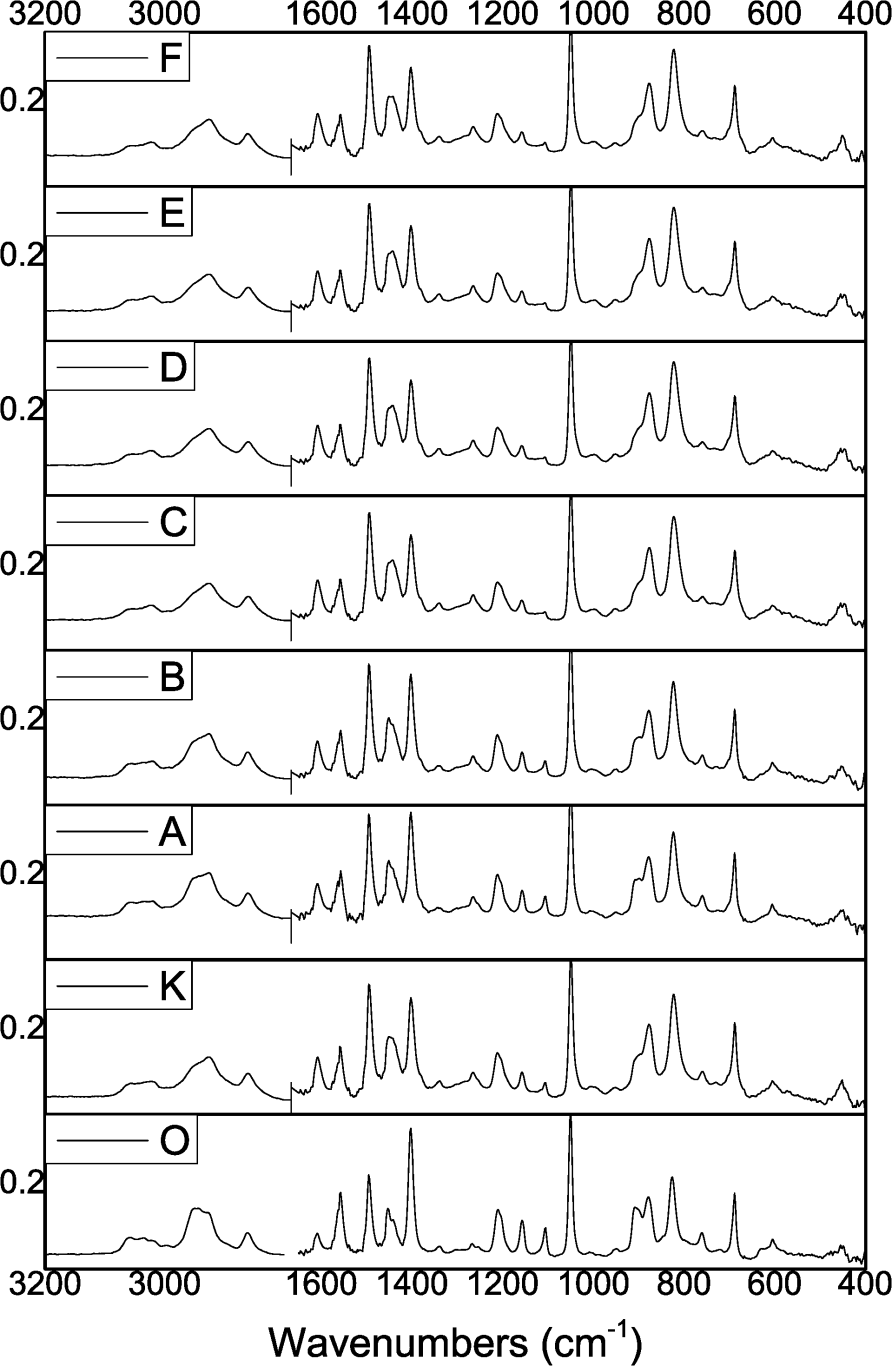
FTIR spectra of pure parylene C (samples O and K) and NCPCs (samples A to F).

According to the FTIR analysis (Figure 5), the main spectral features of parylene C appear in all NCPC samples regardless of Ag content and AgO*_x_* nanoparticle size. Compared to sample O (pure PPXC), neither shift or disappearance of the most intense parylene C peaks nor appearance of new peaks is found in the spectra of sample K and of all the NCPC samples. Only a slight broadening of some specific peaks occurs, especially of the peaks at 3020 cm^−1^ (aromatic C–H stretching), 2950, 2926 and 2861 cm^−1^ (C–H aliphatic stretching of methylene groups –CH_2_), 1452 cm^−1^ (C–H bending), 877 cm^−1^ (one adjacent C–H bending on benzene ring) and 825 cm^−1^ (two adjacent C–H bending on benzene ring) [60]. Moreover, the intensity of some minor peaks slightly changes. It increases for peaks at 1608 cm^−1^ (aromatic C–C ring stretching [57]) and 455 cm^−1^ (out-of-plane ring bending [66]), whereas it decreases for the peaks at 1157, 1106, 908, and 758 cm^−1^. It is noteworthy that all these changes are already visible in the spectrum of sample K (no Ag, plasma on), thus indicating that they are mostly due to the plasma effect and that the incorporation of AgO*_x_* nanoparticles plays a less important role. The persistence of the main spectral features and the limited changes of the abovementioned peaks lead to rule out a damaging effect of both plasma and nanoparticle incorporation on the parylene chains, with the formation of molecular fragments during the deposition process. Instead, it is inferred that they can be related to the change of the crystalline structure of the parylene matrix in the plasma-deposited samples, as already pointed out by the XRD analysis, consisting in a decrease of the preferred orientation of parylene nanocrystallites. As a matter of fact, the surrounding chemical environment of any molecule affects the IR activity of its vibrational modes (i.e., the changes of the dipole moment as induced by IR radiation absorption). Therefore, the different proximity of parylene chains with nearby chains and AgO*_x_* nanoparticles due to different structural arrangements can give rise to the effects observed in our samples. A similar behavior was also found in parylene C samples doped with an UV-absorbing compound [56].

### Dielectric analysis: motivation for OFETs

For OFET applications, parylene C is often selected due to its numerous advantages, as clearly highlighted in a recent paper on the subject [3]. As discussed in the Introduction, one motivation here to develop NCPCs is the integration as gate insulating layer in such applications. A sufficiently high capacitance *C*_i_ of the gate insulating material is required for optimizing performances in OFETs [3]. *C*_i_ is given by:

[1] $$C_{\text{i}}=\frac{\varepsilon_{0}\varepsilon^{\prime }}{t}S\;,$$

where ε_0_ is the vacuum permittivity (8.85 × 10^−12^ F·m^−1^), *t* the thickness of the dielectric, *S* the surface of electrodes and ε’ (often named *k* in the industry of microelectronics) is the dielectric constant (more rigorously called relative permittivity). As seen by this equation, the insulating gate capacitance *C*_i_ is directly proportional to ε’. Typically, for parylene C ε’ = 3.15 (at 1 kHz and room temperature [34]) and an increase of this value will have a direct positive repercussion on the efficiency of the field effect.

We have developed our parylene stacks with a view to propose a new approach and a compromise to the solutions provided so far. The parylene layer doped with AgO*_x_* nanoparticles increases the dielectric constant and responds to the increase in performance given in Equation 1. In order to maintain the good insulator/semiconducting interface and for keeping good band structures at the gate–insulator interface, the AgO*_x_*-containing parylene C is encapsulated by undoped parylene C (samples A to F). In order to avoid an increase of the gate voltage to control the channel, we had the concern to keep a total thickness of our stack of the same order of magnitude as a single layer of parylene commonly encountered in applications. Thus, as reported in Table 1, thicknesses are in the range of 2 ± 1 µm.

**Figure 6:**
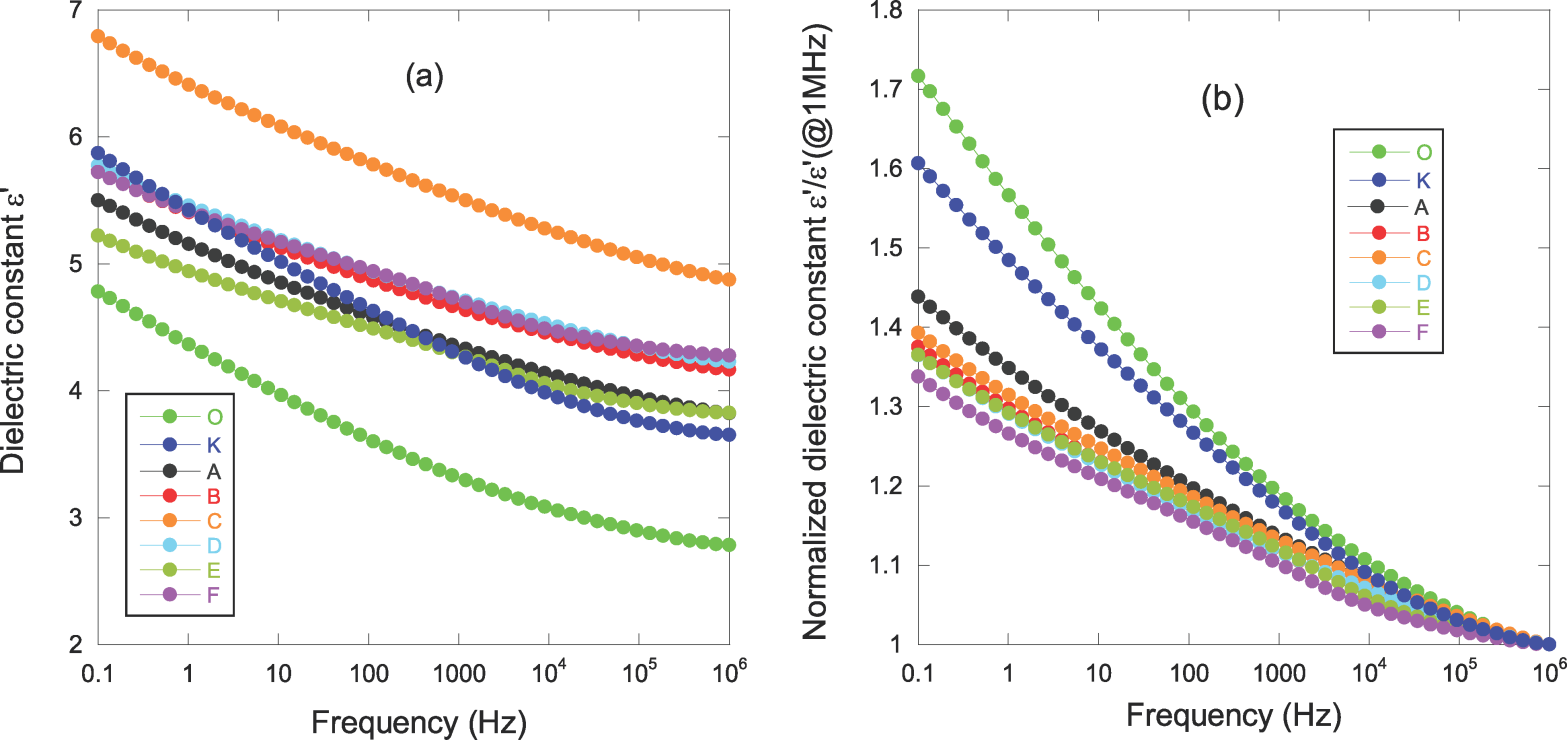
a) Dielectric constant ε’ as a function of the frequency; b) normalized dielectric constant ε’/ε’_HF_ (@1 MHz).

Figure 6a reports the frequency dependence of the dielectric constant for pure parylene (samples O and K) and NCPC films (samples A to F).

The general observation of an increase in the dielectric constant ε’ with a decrease in frequency was clearly explained by the dipolar relaxation of the C–Cl bond (β-relaxation) [34]. Compared to the pure parylene sample (O) all other samples present a higher dielectric constant over the whole frequency range. Many factors could explain this result:

1. An increase in polymer thickness sometimes leads to an increase in the dielectric constant. This was observed for example in polyimide [67] and parylene C [65] films. It is attributed to the interaction between the polymer chains and the substrate and also to the orientation of polymer chains along this substrate. In our case, the change in the value of the dielectric constant is too big to consider such mechanisms.

2. The influence of the plasma must be taken into account as a significant effect. When comparing samples O and K, the plasma induces a mean ε’ increase of 0.98 ± 0.12 over the whole frequency range. Referred to GIXRD analyses, sample K presents a higher degree of crystallinity than sample O, as highlighted by the shift of the (020) peak to a larger 2θ angle (Figure 3, left) and by the larger nanocrystallite size (reduction in the FWHM, see Table 2). Typically, when comparing two similar polymers with just a change in the degree of crystallinity, the most important parameter modifying the frequency trend of the β-relaxation is the dielectric strength Δε = ε’_LF_ − ε’_HF_ [62], where ε’_LF_ is the low-frequency dielectric constant (measured at 0.1 Hz in our case) and ε’_HF_ is the high-frequency dielectric constant (measured at 1 MHz in our case). Δε is associated to the number of dipoles participating in the β-relaxation process: the higher the degree of crystallinity for a given polymer, the lower the number of dipoles involved in the relaxation mechanism (as these dipoles are ‘frozen’ in the semi-crystalline state). Consequently, Δε decreases at increasing degrees of crystallinity [68]. For our results, Δε_sample O_ = 2 and Δε_sample K_ = 2.2. Moreover, as mentioned above, ε’_sample K_ > ε’_sample O_ over the whole frequency range. Both results do not agree with the previous statement and another explanation must be explored.

As shown by RBS investigation, a decrease of film density and a larger amount of parylene are obtained when the plasma is switched on. The larger amount of parylene is accompanied by a larger amount of C–Cl bonds, which will then concur to increase both ε’ and Δε. The β-relaxation is a local phenomenon and is expected to be little affected by the density of the film. Hence, we conclude that a greater amount of parylene explains the difference in ε’ behavior between samples O and K. If one normalizes ε’ to ε’_HF_ (Figure 6b), one can see that Δε_normalized_ = Δε/ε’_HF_ is lower for sample K. The higher degree of crystallinity makes less C–Cl dipoles (in percentage) participate in β-relaxation.

3. Comparing all samples subjected to plasma, GIXRD reveals that the degree of crystallinity and the parylene crystallite size are not very different in the NCPC samples. Therefore, the origin of the difference in the dielectric behavior between all these samples has to be found in the presence of AgO*_x_* nanoparticles in the parylene matrix. The incorporation of conductive particles into a dielectric matrix can lead to a consequent increase in the dielectric constant due to the high polarizability of these conductive particles [69]. A moderate amount of Ag-containing nanoparticles and the fact that these particles are in an oxide phase are the most likely cause of such a small change in the value of the dielectric constant in our present work. However, the values and frequency-dependence of ε’ do not seem to follow a coherent trend as a function of the Ag content and, at first sight, irregular behavior appears.

**Figure 7:**
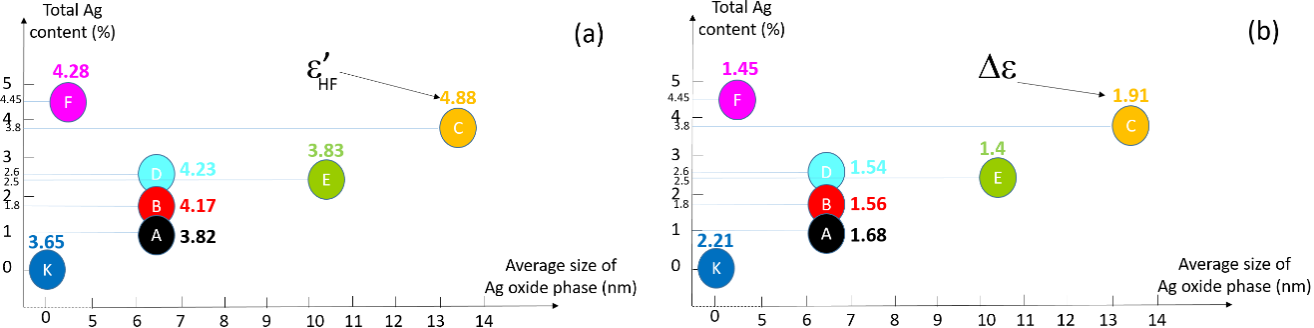
The amount of silver-oxide nanoparticles as a function of their average size. Additional information next to the sample symbol and in the same color of sample symbol: a) the high-frequency dielectric constant ε’_HF_ (value at 1 MHz); b) the dielectric strength Δε.

To better clarify the influence of AgO*_x_* nanoparticles on the dielectric response of NCPCs, let us first focus on the value of the high-frequency dielectric constant ε’_HF_. No strict correlation appears between Ag content and ε’. Indeed, the sample C with Ag content of 3.8% presents the highest dielectric constant; samples B, D, F with respective Ag contents of 1.8%, 2.6%, 4.45% have similar dielectric values. Consequently, another parameter influences the results. In order to help the analysis of the results, we positioned the different samples on a graph in *x*-coordinate representing the average size of the Ag oxide nanoparticles and in *y*-coordinate representing the total amount of silver (Figure 7).

In this graph, we show for each sample the value of the high-frequency dielectric constant ε’_HF_ (Figure 7a) and the dielectric strength Δε (Figure 7b). As highlighted in Figure 7a, at a given average size of Ag oxide particles, ε’_HF_ increases with higher Ag content (comparison of samples A, B, D). A very low size of Ag oxide particles (≤5 nm) combined with a high Ag content increases ε’_HF_ very little (sample F compared to sample D). However, a combination of high Ag content and high average size of Ag oxide nanoparticles is expected to give rise to an improved ε’_HF_ as shown by sample C. Intermediate values of both Ag content and AgO*_x_* size are not beneficial to obtain high values of ε’_HF_ (sample E).

Let us now focus on the frequency-dependence of the dielectric constant ε’. Using Figure 6b, we can clearly see that the presence of AgO*_x_* nanoparticles induces a weaker Δε_normalized_ (38 ± 5%) than in the samples without nanoparticles (60% for sample K and 70% for sample O). This reflects that AgO*_x_* nanoparticles generate chain entanglement or crosslinking of the polymer chains thus decreasing the cooperative motion of chains and causing a reduction in the dielectric strength of the β-relaxation. An analogy to β-relaxation can be made by saying that the addition of AgO*_x_* nanoparticles behaves as an overall increase in material crystallinity (i.e., Δε_normalized_ decreases). This analogy is emphasized when one compares sample K (absence of nanocomposites) to NCPCs (Figure 6b and Figure 7b). Clearly, Δε is higher revealing a lower global ‘semi-crystalline state’ for sample K. From Figure 7b, at a given average size of Ag oxide phase (samples A, B, D), Δε is reduced with higher Ag content, which is in line with our argument that adding nanoparticles effectively reduces the mobility of polymer chains. Comparing samples E and F (Figure 7b), they present a similar Δε, but the former has a lower amount of silver-oxide nanoparticles with a larger average size. Sample C stands out once again with a larger Δε than the other NCPCs. This last result seems surprising and contrary to our hypothesis of polymer chains restricted by the addition of nanoparticles and their size. However, let us not forget that the sample C has the greatest thickness (see Table 1) and therefore contains a larger ‘reservoir’ of dipoles available to participate in β-relaxation. TEM analysis should be carried out to assess the volume occupied by nanoparticles and possible agglomerations of AgO*_x_*. This characterization was beyond the scope of this study but would deserve particular attention for future work.

**Figure 8:**
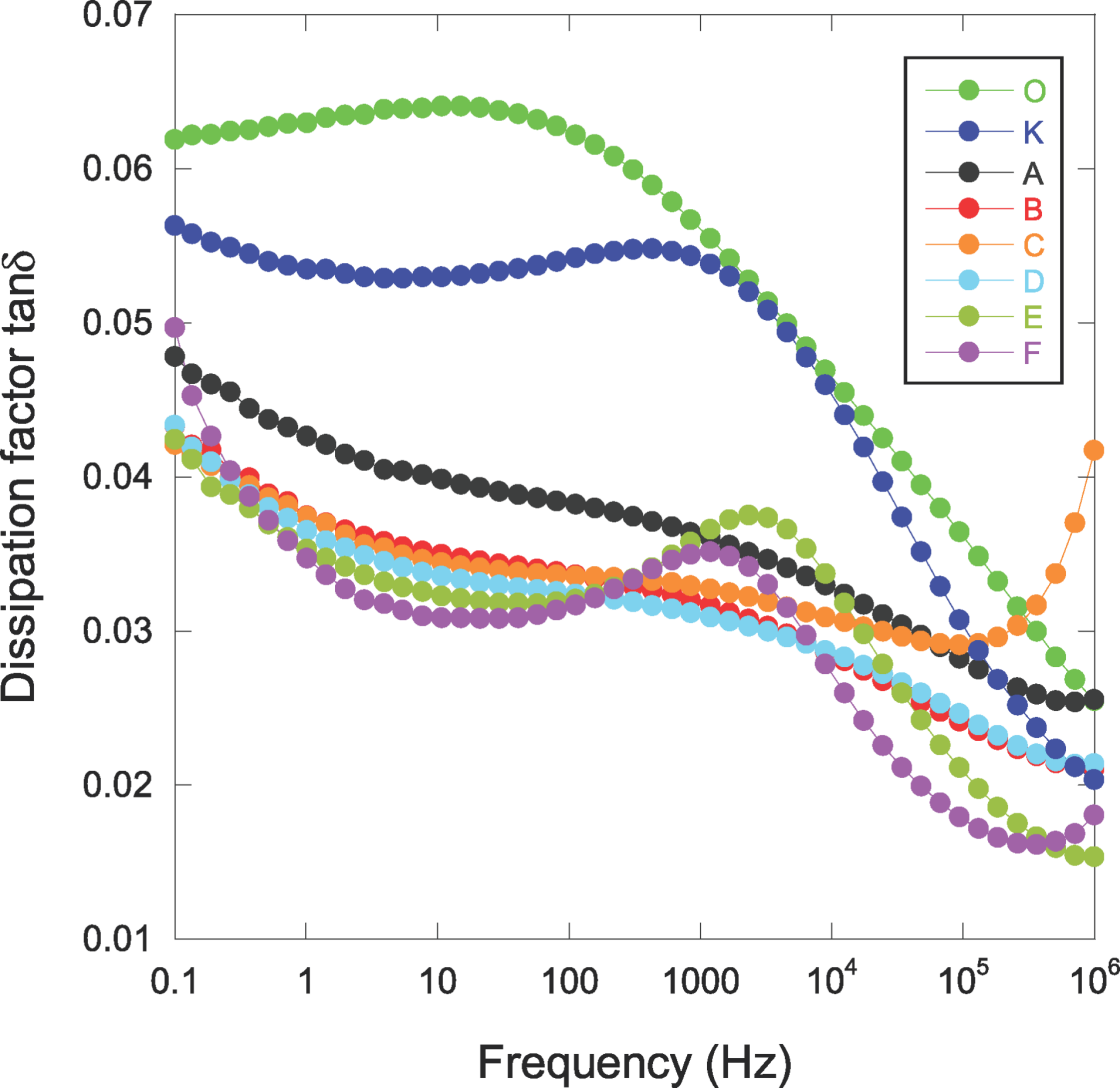
Frequency dependence of the dissipation factor for pure parylene C and NCPC samples.

It is well known that the addition of particles with a high dielectric constant to a polymeric material causes (as generally desired) an increase in the dielectric constant but is also accompanied by an unwanted increase of dielectric losses (imaginary part of the permittivity ε” and the dissipation factor tan δ = ε”/ε’), which limits their integration for the envisaged application. We have evaluated these losses in our materials and the results are shown in Figure 8.

Not surprisingly, the appearance of a broad peak over the entire frequency range for sample O is representative of β-relaxation [34]. The effect of plasma (sample K) results in a slight decrease in these losses over the entire frequency range. However, there is a slight increase in these losses at the lowest frequencies (less than 1 Hz) attributed to the manifestation of charge conduction or a new polarization mechanism. A temperature study would make it possible to decide on this rise.

It is worth noting that the addition of silver-oxide nanoparticles leads to a reduction in tan δ. Sample C, which has the highest dielectric constant, also shows a low tan δ. The increase in the dissipation factor at the higher frequencies for this sample is probably the consequence of a parasitic impedance at the electrode–polymer interface. This observation is related to the fact that this material has the highest roughness (see Table 2) and the deposition of the upper electrode for the measurement is probably impacted by the roughness.

**Figure 9:**
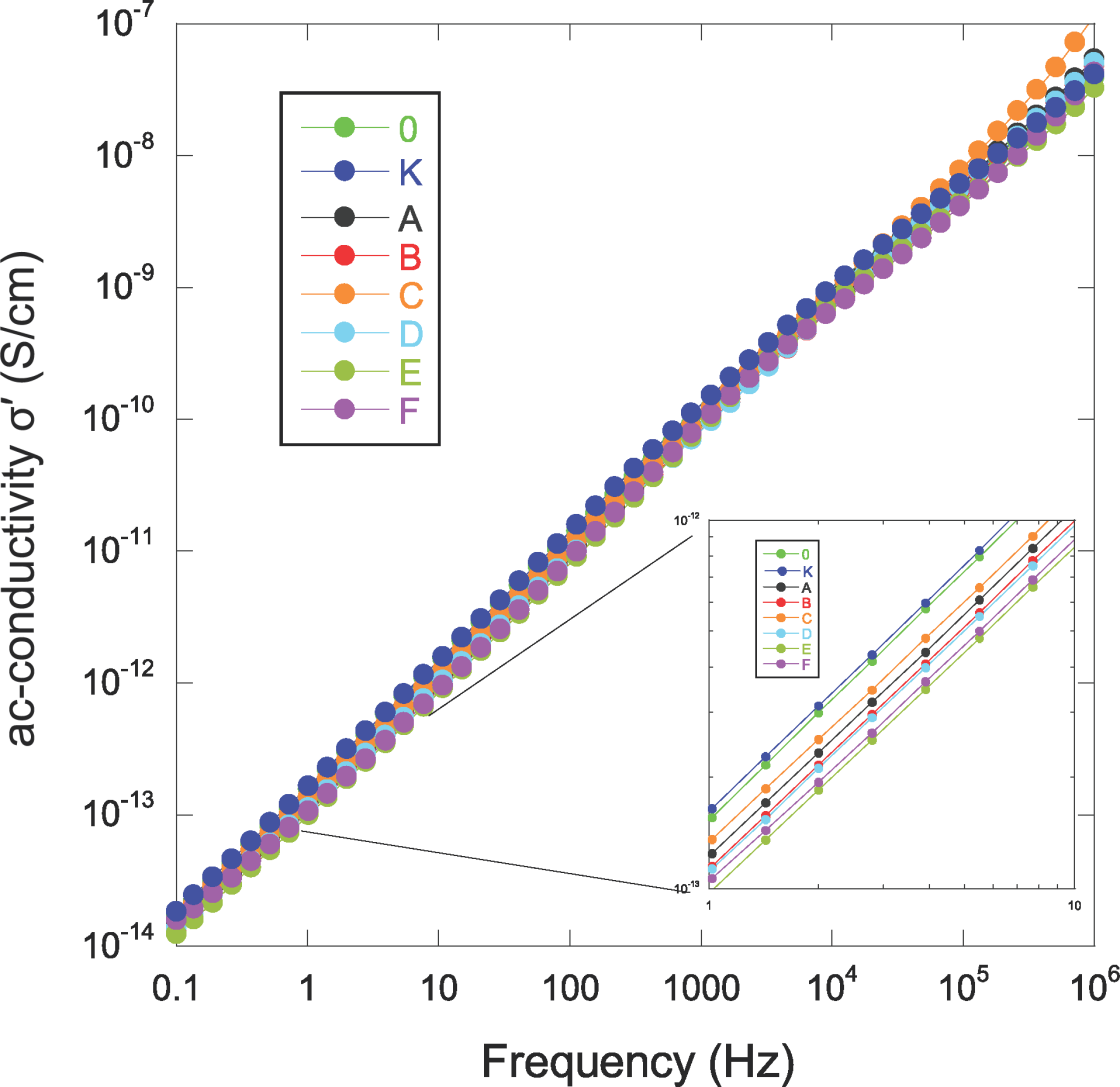
Electrical conductivity of pure parylene C and NCPCs.

The most pronounced effect of a moderate addition of nanoparticles appears to be an increase in the dielectric constant rather than a degradation (increase) in the imaginary part of the permittivity ε”. These results indicate that silver oxide reduces the dielectric strength as related to the β-relaxation without bringing other inconveniences such as interfacial polarization mechanism or a long-range electrical conductivity induced by these nanoparticles. For this latter, we plotted the electrical ac-conductivity σ’ as a function of the frequency (Figure 9).

The behavior is representative of short-range conduction (hopping) without significant effect of Ag nanoparticles besides a weaker improvement in σ’ (see inset in Figure 9). In the range around 1 kHz, one observes a change in the slope of the ac-conductivity for sample F. This specific effect is well highlighted in the tan δ response (Figure 8) with the appearance of a peak for this sample and also for the sample E. It has to be remembered that these two samples have the weakest Δε (see Figure 7b), hence β-relaxation will mask less any other mechanism that may influence the dielectric response (or from another point of view: The other phenomenon drives the mechanism of β-relaxation much more, explaining the low Δε). The tan δ peak observed for the sample F could be justified by the large amount of Ag nanoparticles, while the reason why sample E exhibits similar behavior remains curious and would require additional work to be completely understood.

**Figure 10:**
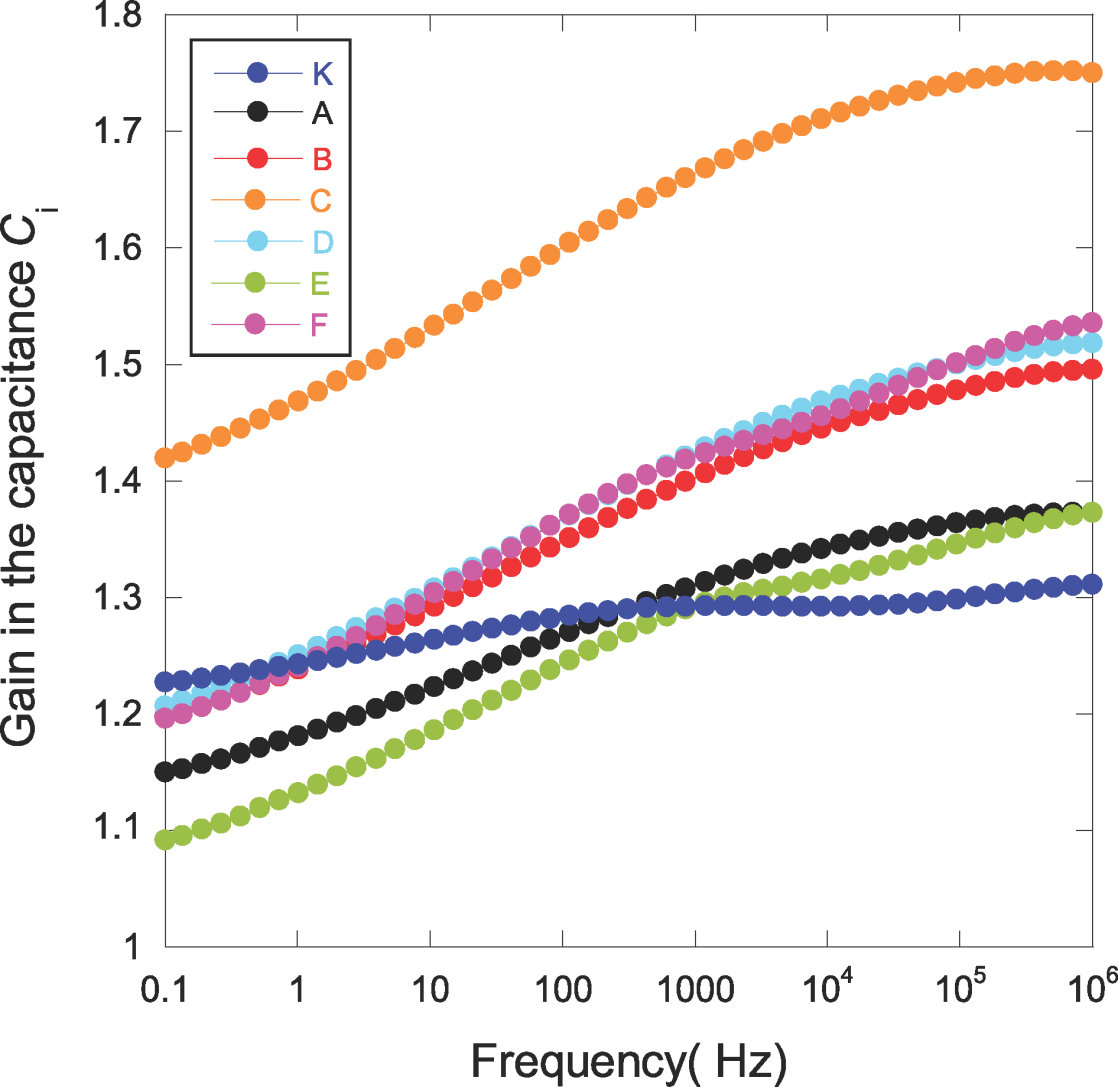
Gain in the gate insulation capacitance by replacing conventionally processed parylene C (sample O) with plasma-combined CVD process incorporating silver-oxide nanoparticles. Accuracy in the gain due to geometric dimensions of samples is in the range ±0.1 (sample F) to ±0.18 (sample C).

This dielectric analysis showed that an increase in the dielectric constant accompanied by a decrease of dissipation factor tan δ is possible when silver-oxide particles are embedded in a parylene C matrix. Thus, for OFET applications, NCPC thin films could constitute a new interesting route as insulating layer. Let us take Equation 1 and Figure 6a. To evaluate the improvement brought about by the addition of silver-oxide nanoparticles in the performance of the grid oxide for OFETs, we standardized (at each frequency) the curves in Figure 6a by the value of the dielectric constant obtained from the sample O (this latter uses a conventional deposition process for the growth of parylene C). Results are reported in Figure 10. Compared to the sample K (no Ag content), samples A and E have no advantages in terms of performance for *C*_i_. Sample C has the highest insulating gate capacitance. However, an improvement in the roughness for this sample must be reached before integration for OFET as this structural parameter is critical in the deposition of organic semiconductor layers and will affect the performance [70–71].

## Conclusion

Nanocomposite–parylene C (NCPC) were synthetized at room temperature by chemical vapor deposition polymerization of parylene C combined with RF-sputtering of silver. It was demonstrated that the plasma itself induces changes in the density and semi-crystalline character of parylene C. A decrease in the density, an increase in the degree of crystallinity and an increase of about 60% in the size of parylene nanocrystallites were observed. The addition of silver-containing nanoparticles does not further modify the crystallinity of parylene C itself. These nanocomposites consist of a silver-oxide phase embedded inside the parylene C matrix. No trace of pure metal silver is found in all the NCPC samples. The roughness and the dielectric properties of NCPC are significantly influenced by the presence of silver-oxide nanoparticles. The roughness increases from 10 nm (for undoped parylene C films) to values from 20 to 160 nm for NCPC, depending on the film thickness, size of silver-oxide nanoparticles and, to a lesser extent, Ag content. The dielectric performance appears suitable for the integration of these NCPC as gate insulating materials for OFETs. Simultaneously, it is possible to increase the dielectric constant (by a factor of 1.4) and to reduce (or to not degrade) the dissipation factor (tan δ < 0.05). Finally, the interest of NCPCs was proved for applications such as integration in OFETs, but improvements in the deposition process should be pursued. The possibility to tune the parameters of the CVD and plasma processes will allow for better controlling the semi-crystalline character of parylene C, the roughness, the thickness of the layers, the amount and the size of nanoparticles. Concerning possible aging effects on the properties of these NCPCs, the stability of both parylene C and silver oxide is a good premise for the achievement of a stable nanocomposite material. However, a specific study of this aspect will be carried out in the future.

## Experimental

### Parylene C/Ag nanocomposite deposition

To avoid the synthesis of the polymer nanocomposite in two stages, we propose a new clean method to simultaneously deposit both the organic compound (parylene C) and the inorganic compond (silver-containing nanoparticles). This method consists of two associated processes, i.e., primary vacuum-CVD and RF-magnetron sputtering.

**Figure 11:**
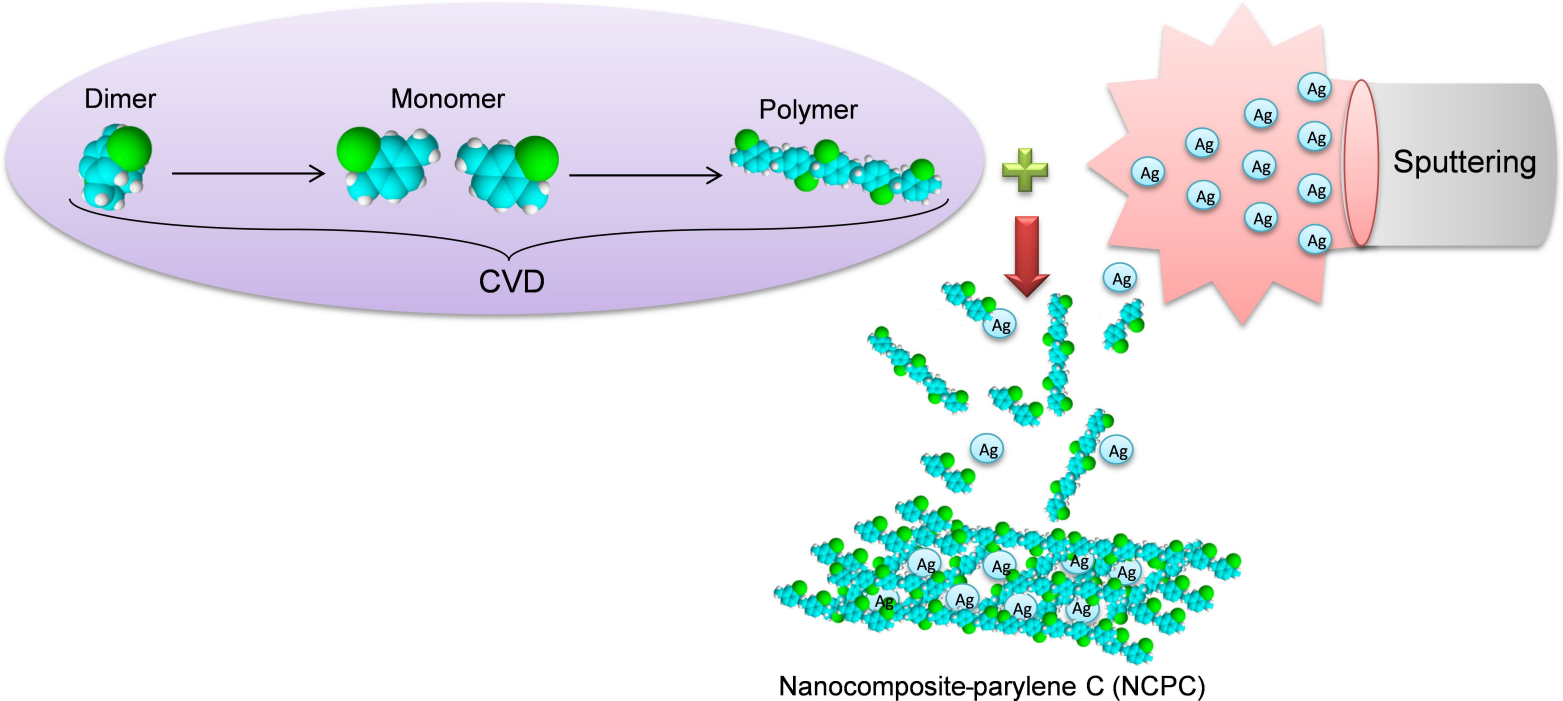
Depostion of nanocomposite–parylene C (NCPC) by a combined CVD and RF sputtering technique.

The deposition process of NCPC involves three successive operations. The process begins with the deposition of parylene C. Some hundreds of nanometers of parylene C film are deposited firstly as an electrical passivation layer onto the metallic substrate (silicon, gold or aluminium). During this step, the RF-magnetron sputtering source is turned on in order to avoid the deposition of parylene C on the Ag target surface, but a shutter is placed between target and substrates to avoiding unwanted incorporation of Ag atoms in the deposited films. Then, the shutter is opened and the parylene C and the silver atoms are deposited at the same time in order to constitute the nanocomposite films (Figure 11). As a final step, the shutter is closed again leaving only the deposition of parylene C as a capping layer between the nanocomposite and the top metallic electrode or even as a coating layer for the nanocomposite.

Analogous to the description in [26], the parylene C deposition consists of three steps. First, the cyclic dimer (dichlorinated di-*p*-xylylene, 2.5 g, same amount for all the depositions) is sublimated at a temperature between 120 and 160 °C and a pressure of around 1–2 Pa in the first step. Then, the vapor of the dimer is cleaved into a reactive vapor monomer (monochlorinated *p*-xylylene) in a pyrolysis chamber at a temperature between 600 and 700 °C. Finally, the monomer molecules in the gaseous state enter the deposition zone to get deposited on substrates. When the RF sputtering process is enabled (open shutter), the silver atoms condense onto the substrates together with the monomer molecules and are then incorporated in the polymer matrix. Under these conditions, we have prepared uniform nanocomposite thin films with thicknesses from 1 to 3 µm, as measured by a Tencor AlphaStep 200 profilometer. In order to obtain several, nominally identical samples for each deposition run, the substrates were put on a rotating carousel, with the rotation axis parallel to the Ag target surface and with a rotation period of 90 s. The sample holder can accommodate up to ten substrates so that at least two samples from each type were used for the different measurements carried out in this work. Different samples were produced by increasing the number of rotations from 0 to 6, as detailed above (see Results and Discussion). The minimum distance between the Ag target and the sample surface was fixed at 10.5 cm.

### Parylene C/Ag nanocomposite characterization

The silver and chlorine content in the nanocomposite samples was quantified by Rutherford backscattering spectrometry (RBS). Ion beam analyses of the deposited films were performed using a 2.0 MeV ^4^He^+^ beam at the Van de Graaf accelerator at the Laboratori Nazionali di Legnaro, with a scattering angle of 160°. We have to highlight that the Cl content measured with RBS (in atoms·cm^−2^) is directly converted into the amount of parylene (in monomeric units·cm^−2^), since each monomeric unit contains one Cl atom. RBS analysis was performed on coatings deposited on silicon substrates. Taking into account the desorption of Cl-containing species from the film occurring during ion beam analysis [72], RBS spectra were acquired by irradiating different spots of the pristine samples and collecting only 0.2 µC of charge on each spot until a total collected charge of a few microcoulombs was reached. A stylus profilometer (Tencor Instruments, model Alpha-Step 200) was used to measure the film thickness. Grazing incidence X-ray diffraction scans were carried out on a Philips diffractometer on the as-grown samples, using Ni-filtered Cu Kα radiation at 40 kV and 40 mA. The incidence angle was fixed at 0.5° for all the samples. Some spectra were also collected at 1° incidence in order to probe deeper the film structure. The surface morphology was analyzed using a non-contact mode AFM model C-21 (Danish Micro Engineering), mounting a DualScope Probe Scanner 95-50. Capacitance areas were defined in the top NCPC resulting in square 2 × 2 mm^2^ contacts. In order to assure a homogeneous distribution of the potential during the dielectric measurements, top gold electrodes with a thickness of 100 nm were deposited by thermal evaporation with the sample held at room temperature. Chemical composition of pure parylene C films and NCPC thin films was investigated at room temperature by Fourier transform infrared spectrometer (FTIR, Nicolet 380) in reflectance mode at a resolution of 4 cm^−1^ in a wave number range from 400 to 3200 cm^−1^. The spectra were obtained after a previous background subtraction with 32 scans for each sample to remove the contribution of H_2_O and CO_2_ molecules. Dielectric properties were measured using a Novocontrol broadband dielectric spectroscopy (BDS20) impedance meter in the frequency range of 0.1–10^6^ Hz at room temperature.
